# Structure-conditioned self-supervised learning of residue interaction constraints in protein kinases for variant interpretation

**DOI:** 10.1093/bioinformatics/btag487

**Published:** 2026-08-04

**Authors:** Shakiba Fadaei, Fanny S Krebs, Vincent Zoete

**Affiliations:** Computer-Aided Molecular Engineering, Department of Fundamental Oncology, Ludwig Institute for Cancer Research, University of Lausanne, Lausanne, 1015, Switzerland; Computer-Aided Molecular Engineering, Department of Fundamental Oncology, Ludwig Institute for Cancer Research, University of Lausanne, Lausanne, 1015, Switzerland; SIB Swiss Institute of Bioinformatics, Lausanne, 1015, Switzerland; Computer-Aided Molecular Engineering, Department of Fundamental Oncology, Ludwig Institute for Cancer Research, University of Lausanne, Lausanne, 1015, Switzerland; SIB Swiss Institute of Bioinformatics, Lausanne, 1015, Switzerland

## Abstract

**Motivation:**

Protein kinases are key regulators of cellular signaling and are frequently implicated in human diseases. Although kinase domains are structurally conserved, predicting the effects of amino acid substitutions remains challenging as mutations often introduce subtle structural perturbations that are not captured by sequence-based or evolutionary methods. Existing supervised approaches further rely on pathogenicity annotations that are inconsistent across databases, thereby motivating the development of structure-based, label-independent frameworks for mutation effect prediction.

**Results:**

We present a structure-based method using SE(3)-transformers to learn residue compatibility with the local structural environment from experimentally resolved kinase 3D structures. Proteins are represented as atom-level graphs with physicochemical descriptors derived from the CHARMM force field and spatial connectivity. The model is trained on two self-supervised tasks given local structural context: masked residue atom reconstruction and masked residue classification. This formulation enables learning of geometric and physicochemical constraints without relying on pathogenicity labels. Evaluation using reconstruction loss, residue prediction accuracy, and comparison with BLOSUM substitution patterns indicate that the model captures biologically meaningful relationships between residue identity and 3D structural context. We interpret the scores assigned to alternative amino acids as measures of structural fitness, where low-scoring residues are hypothesized to be less compatible with the local environment and more likely to induce deleterious effects on protein structure and activity.

**Availability and implementation:**

https://zenodo.org/records/20393799.

## 1 Introduction

Protein kinases play a crucial role in cellular processes by regulating protein activity, localization, stability, and interactions (Hunter 2000, Pawson and Scott 2005). They exert these functions through phosphorylation, a fundamental regulatory mechanism that controls cellular signaling (Johnson *et al.* 1998, Cohen 2002). Phosphorylation governs essential biological processes including cell growth, proliferation, differentiation, stress responses, and programmed cell death (Hanks and Hunter 1995, Cohen 2000, Johnson 2009). Given their central regulatory roles, alterations in kinase function are strongly associated with human diseases, making kinases one of the most extensively studied protein families (Zhang *et al.* 2009).

Analyzing t3D structures of proteins and underlying physicochemical interactions is essential to understand their functions (Hubbard and Till 2000, Nolen *et al.* 2004, Liao 2007). The kinase domain is highly structurally conserved despite its functional diversity (Taylor and Kornev 2011). Kinases adopt a bilobal structure, composed of an N-terminal lobe and a C-terminal lobe (Huse and Kuriyan 2002, Modi and Dunbrack 2019). The N-terminal lobe is enriched in β-strands while the C-terminal lobe is predominantly composed of α-helices. The two lobes are connected by a hinge region, where the ATP-binding site is located (Knighton *et al.* 1991, Endicott *et al.* 2012). Mutations in the kinase domain can influence the atomic interactions, altering its conformation or fold (Saladino and Gervasio 2016). Understanding how individual amino acid substitutions affect kinase function remains a major challenge, as mutations can alter protein behavior through subtle structural perturbations that are not readily captured by evolutionary conservation alone (Izarzugaza *et al.* 2011, Dixit and Verkhivker 2014). Predicting the impact of such mutations therefore requires models capable of capturing structural context rather than relying solely on sequence information (Saladino and Gervasio 2016, Rodrigues *et al.* 2018). Deep learning structure predictors that rely on sequences tend to preserve the global fold when modeling point mutations. This happens even in some cases where experimental evidence indicates deleterious effects of mutations. Thus, such models are often insufficient for predicting point mutation effects (Garrido-Rodríguez *et al.* 2024).

Recent advances in geometric deep learning enable protein structures to be modeled directly as 3D objects while respecting physical symmetries such as rotational and translational invariance. These approaches provide a natural framework for learning how local atomic environments constrain residue identity and compatibility within a folded protein structure (Krapp *et al.* 2023).

In this work, we present a structure-based framework for predicting mutation effects in protein kinases using SE(3)-transformers, which are equivariant to translations and rotations, applied to experimentally determined protein structures (Fuchs *et al.* 2020). Protein structures are represented as graphs, where nodes correspond to atoms characterized by CHARMM force fields and their mass, and edges encode spatial relationships between neighboring atoms (Huang and MacKerell 2013). During training, residues are masked, and the model reconstructs the atoms of the hidden residue using only its *n* nearest neighboring atoms in 3D space. By solving this reconstruction problem, the model learns the geometric and physicochemical constraints that determine residue compatibility within a given structural environment. The model assigns a score to each of the 20 canonical amino acids as the prediction for the masked residue. We hypothesize that these scores reflect the local structural fitness at a specific position. Residues with high reconstruction likelihood are predicted to be well accommodated within the local environment and are therefore more likely to have no or minimal structural impact (predicted as neutral), whereas residues assigned low scores are expected to be structurally damaging and more likely to affect the kinase activity (predicted as damaging). Framing mutation effect prediction as a structure-conditioned residue reconstruction task provides a self-supervised framework that does not require pathogenicity labels during training. Currently, training supervised models using pathogenicity labels is not ideal, as available pathogenicity data are highly heterogeneous and often incoherent across databases. This inconsistency limits their reliability for a binary classification task distinguishing neutral from damaging variants.

By integrating experimentally resolved kinase structures with equivariant deep learning, this approach captures fine-grained structural constraints governing mutation tolerance. It also provides a general framework for interpreting genetic variation within structurally conserved protein families.

## 2 Materials and methods

### 2.1 Data extraction

#### 2.1.1 PDB structures

The list of kinases, including UniProt identifier (IDs) and kinase domain range, was extracted from KinCoRe, a structure-based multiple sequence alignment (MSA) resource comprising 497 kinases. 7410 kinase structures corresponding to these UniProt IDs were then retrieved from the Protein Data Bank (PDB) (Berman *et al.* 2000). By aligning each PDB structure sequence with its UniProt sequence, we determined 3301 correspond to the wild-type protein. The remaining structures corresponded to mutant variants.

#### 2.1.2 Annotated variant data

The aim of the project is variant interpretation. To achieve this, we have gathered available annotated variant data from NeXtProt (Gaudet *et al.* 2017), GnomAD (Gudmundsson *et al.* 2022), COSMIC (Tate *et al.* 2019), dbCPM (Yue *et al.* 2020), dbSNP (Sherry *et al.* 2001), UniProt (UniProt Consortium 2023), and ClinVar (Landrum *et al.* 2020).

### 2.2 Graph representation

The structures were described as graphs where each node corresponded to an atom. Atom descriptors included physicochemical parameters derived from the CHARMM force field (partial atomic charge, as well as Lennard-Jones parameters Rmin and epsilon), normalized atomic mass and B-factor. The edges of the graph were defined by the pairwise 3D distances between atoms. The structural data was partitioned into training, test, and validation sets. The model operates as an *n* nearest neighbor graph model, where *n* = 16, 32, and 64. This allows the model to operate on local subgraphs instead of the full structure input. To generate the masked partial graphs, we first identified all the *n* nearest atoms to each atom of the residue we wanted to mask. We then provided the node features of the surrounding atoms, and the connectivity between them. No information—neither node features nor connectivity—pertaining to the masked atoms was included in the input. The data points were partial graphs that each had one masked residue. We generated partial graphs masking each position, resulting in 2 269 125 partial graphs; where 1 488 792 are used for training, 527 761 for validation, and 252 572 for the final test set.

### 2.3 SE(3)-transformer model

We employed an SE(3)-transformer to model protein structures as atom-level graphs, explicitly incorporating their 3D coordinates and atom properties. SE(3)-transformers are geometric deep learning models well-suited for 3D data. The group SE(3) represents rotations and translations, in 3D. Equivariance to SE(3) means that if an input structure is rotated or translated, the model’s output transforms consistently rather than changing arbitrarily, making it ideal for modeling molecular systems. By combining attention mechanisms with equivariant feature representations, SE(3)-transformers can learn spatial relationships between atoms in a physically meaningful way (Fuchs *et al.* 2020). Specifically, we used a masked residue prediction setup in which each residue is iteratively hidden and the network reconstructs its atomic positions while identifying the most compatible amino acid based on its *n* nearest neighbors. Atom-level features allowed the model to capture both the local chemical environment and the broader structural context critical for assessing mutation effects. The SE(3)-transformer model was trained to perform two tasks simultaneously: (i) to reconstruct the full local structure from the masked input, including the node features and connectivity information of both the masked and surrounding atoms and (ii) to predict which of the 20 canonical amino acids corresponds to the masked residue.

### 2.4 Hyperparameter optimization

The complexity of both the model and the data led to many hyperparameters that require tuning and exploration. These hyperparameters determine the width and depth of the model. To avoid overfitting, we had to balance the total number of trainable parameters in the model versus the size of the training dataset. Conversely, the model also had to possess sufficient capacity to learn the complex structural data, for which an adequate number of trainable parameters was necessary. The hyperparameters that we optimized were batch size, number of layers, number of attention heads, number of fibers, and learning rate.

### 2.5 Model evaluation

The first evaluation step in model optimization was inspecting the loss trajectory. The model performed two simultaneous tasks: (i) reconstructing the full partial graph from a masked partial graph, for which the loss was measured using the mean squared error (MSE) and (ii) masked residue prediction, a multiclass classification task where the loss was measured using the categorical cross-entropy. The resulting loss trajectories were examined to ensure the absence of overfitting or underfitting. Ideally, the training loss should decrease steadily and should eventually plateau without drastic fluctuations, while the validation loss should follow a similar downward trend. If the training loss improves but validation starts increasing, this is a sign of overfitting. If both losses stay high without decreasing, then the model is underfitted. A good model should generalize effectively, capturing the underlying patterns of the data without fitting to noise.

Moreover, we monitored the accuracy of the masked residue prediction model. The model was primarily informative not for recovering the masked residue—which was already known—but for what it had learned about structural constraints and the scores it assigned to all possible amino acids at a given position. We hypothesized that residues with the lower scores exhibit reduced structural fitness within a given environment, and that substitutions resulting in such residues are therefore more likely to be structurally damaging. We also compared the residue confusion matrix with BLOSUM (BLOcks SUbstitution Matrix), to assess if the scores generated by the model reflect biologically meaningful relationships rather than random fluctuations. BLOSUM is a scoring matrix used to evaluate how likely one amino acid is to be substituted by another during evolution. The comparison was made to BLOSUM45, 50, 62, 80, and 90, which are thresholds used to cluster proteins based on sequence identity. The lower numbers are for more divergent sequences and higher numbers are for more closely related sequences (Henikoff and Henikoff 1992). Due to high structural similarity in protein kinases, such models have a strong potential of overfitting the input. Even with a good performance on the validation set, the partial graphs could have high similarities despite being split to train, validation, and test sets in the first steps, before partial graph construction. To study the model’s ability to overfit the data, after achieving an acceptable model, we trained the same model with shuffled residue labels. If a model has the ability and number of parameters to overfit, it should learn or “memorize” the shuffled labels on the train set. This way the model cannot be reliable (Zhang *et al.* 2016). Ultimately, we assessed the model performance on another protein family, i.e. the small GTPase, which was not presented to the model during the training phase. The goal was to explore if the model has truly learned the underlying atomic interaction constraints or has just memorized the labels.

## 3 Results

### 3.1 Model architecture and optimized hyperparameters

**Figure 1:**
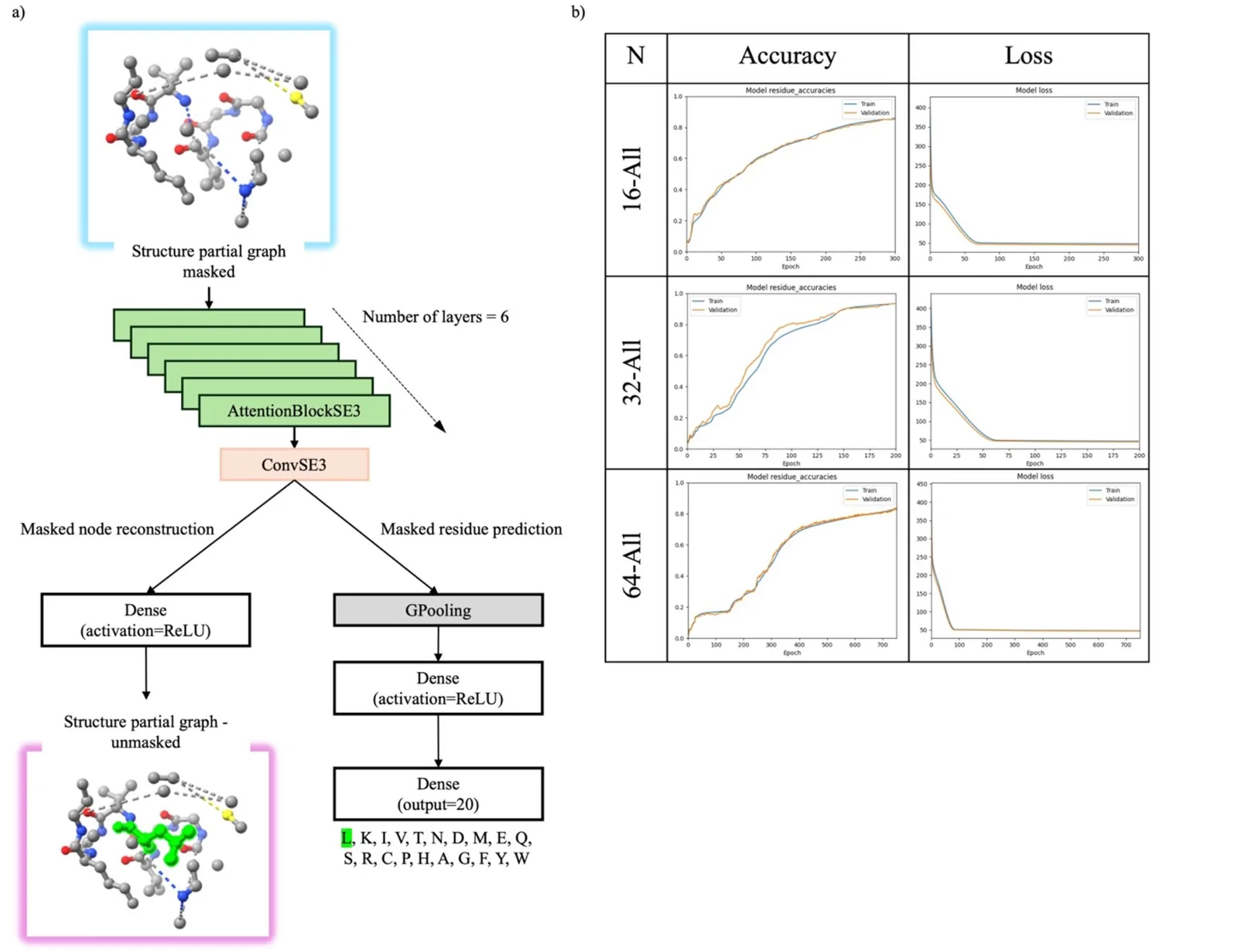
SE(3)-transformer model architecture and performance. (a) The architecture of the masked node reconstruction and masked residue prediction models. The input is masked partial graphs of the structures. (b) The loss and accuracy plots of the trained models for validation and training sets. The models are trained for 16, 32, and 64 neighbors on all structures, including wild-type and mutated ones.

The model requires sufficient depth to capture the complexity of protein structures at atomic level representation. However, increased depth constrains the permissible width of the network, since the total number of trainable parameters should remain commensurate with the number of available data points. We tested 3 to 15 number of layers and found that 6 layers constitute an optimal depth for this task. It has been shown that model depth helps capture abstract patterns. Model depth higher than 6 led to an unstable model, where the loss is infinite. This instability is a common issue with SE(3)-transformers and deeper models (Fuchs *et al.* 2020). To overcome this issue, we implemented optimization strategies, including gradient clipping, lowering the learning rate, and using skip connections. However, it did not result in stable models for numbers of layers higher than 6. The model architecture is shown in Fig. 1a. We trained models on *n* = 16, 32, and 64 neighbors around the masked residue atoms. We additionally trained models using either only wild-type structures or the full dataset of structures, including the mutated ones.

### 3.2 Annotated variant data

We parsed and analyzed the mutation data from NeXtProt, UniProt, GnomAD, COSMIC, dbCPM, dbSNP, UniProt, and ClinVar (Sherry *et al.* 2001, Gaudet *et al.* 2017, Tate *et al.* 2019, Landrum *et al.* 2020, Yue, Zhao, and Xia 2020, Gudmundsson *et al.* 2022, UniProt Consortium 2023). Table 1 reports the number of annotated missense variants in each database, the variants occurring in a protein containing at least one kinase domain, and the variants occurring in the kinase domain. We further report, in the column labeled “consistent”, the subset of annotated mutations that are (i) consistent with their corresponding kinase sequence and (ii) coherent across different databases. Importantly, this column reflects only the absence of explicit conflict between databases; thus, this could mean that this annotation only occurred in one database. This table highlights the substantial noise present in currently available mutation annotations. For example, out of 80k mutations reported by COSMIC within kinase domains, only 44k are consistent. Thus, nearly half of annotated variants exhibit inconsistencies. This proportion exceeds half for GnomAD. For a binary annotation task (neutral versus damaging) such a degree of inconsistency does not merely introduce noise but effectively approaches randomness. Consequently, these data are unsuitable for training supervised mutation prediction models, which motivated our reliance on self-supervised models grounded in experimentally resolved and reliable structural data.

**Table 1:** Annotated mutation data across databases.^a^

Database	Missense variants	Kinase protein	Kinase domain	Consistent
GnomAD	∼19M	∼6k	409	169
COSMIC	∼11M	∼300k	∼80k	44 496
dbSNP	∼125k	∼8k	∼1.7k	1504
UniProt	∼80k	∼7k	∼1.5k	1343
ClinVar	∼20k	900	203	194
NeXtProt	∼9k	361	176	176
dbCPM	∼1.6k	391	129	84

a The number of all annotated missense variants and the number of missense variants found in the kinase domain are shown. The “Consistent” column reports the number of mutations within the kinase domain that have not been annotated differently in other databases. Yet, this category also includes mutations that are not annotated in any other database. Therefore, consistency alone does not necessarily imply agreement across all databases.

### 3.3 Model evaluation

#### 3.3.1 Accuracy and loss trajectory

The first evaluation step in model optimization is the inspection of the training and validation loss trajectories. The accuracy and loss of the models are shown in Fig. 1b. These plots are presented for models using *n* = 16, 32, and 64 nearest neighbors, trained on all structures including the wild-type and mutated ones. Figure S1, available as supplementary data at *Bioinformatics* online shows the loss and accuracy of the models trained only on wild-type structures for 16 and 32 neighbors. The losses shown are the sum of the losses of the two simultaneous tasks: MSE for partial graph reconstruction and categorical cross-entropy for residue prediction. To monitor the performance on the residue prediction model, we also display the prediction accuracy of the models in Fig. 1b. For all values of *n* and models that are trained on both wild-type and all structures, the loss trajectories do not exhibit signs of overfitting or underfitting. The validation loss closely follows the training loss, indicating stable training dynamics and good generalization performance. Moreover, the masked residue prediction task achieves high accuracy, above 80%, across all models.

#### 3.3.2 Model reconstruction success rate

**Figure 2:**
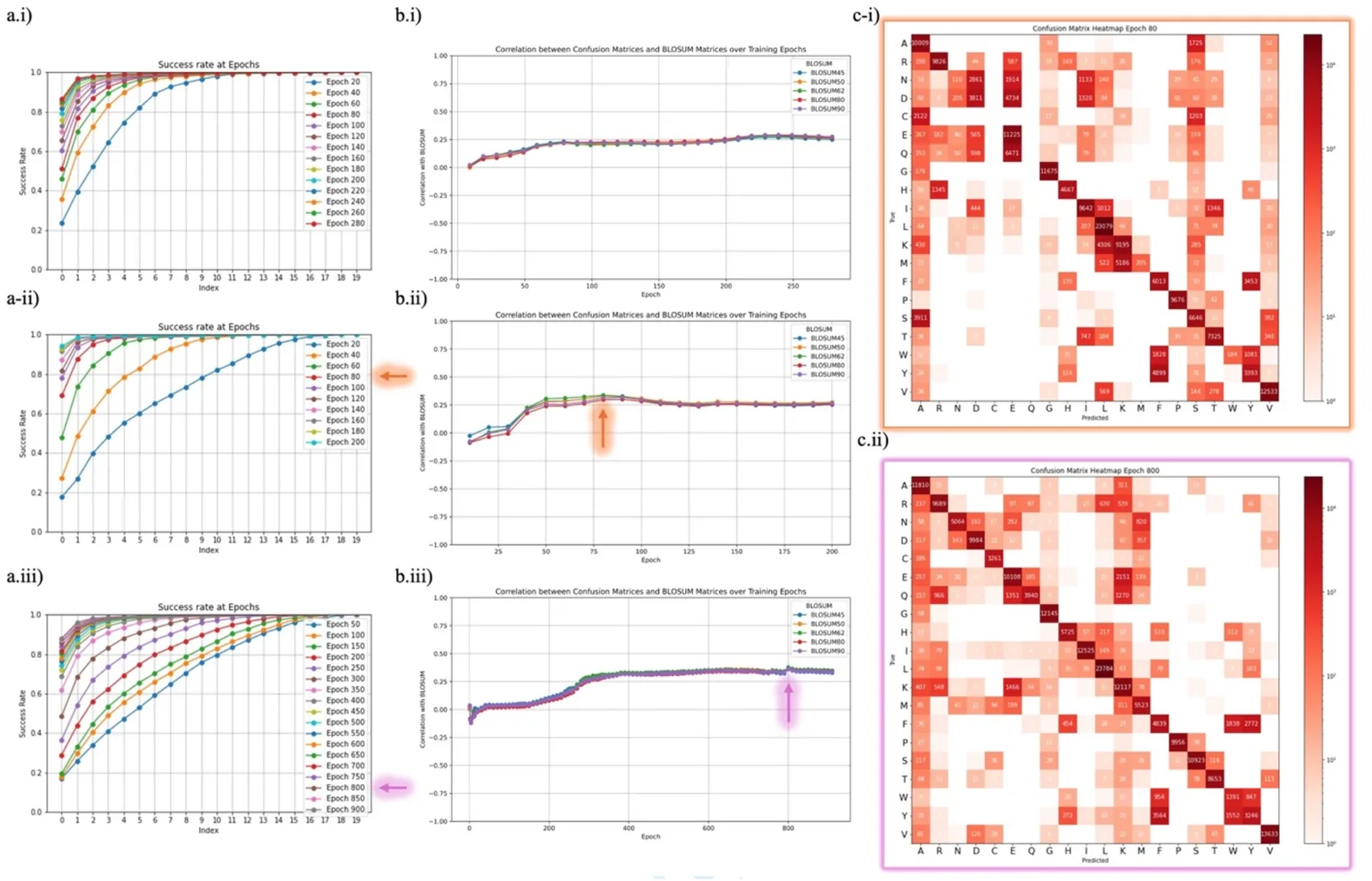
Model performance evaluation. (a) The success rate of the trained models during training epochs. (b**)** Correlation between the confusion matrix and BLOSUM matrix. (i–iii) are for 16, 32, and 64 neighbors. (c) The confusion matrix of the models at the epoch with the highest correlation with BLOSUM matrices for (i, ii) 32 and 64 neighbors.

We measured accuracy as an initial metric for model evaluation. Accuracy is defined based on the top prediction of the model. Another informative metric is the success rate of the model, which measures if the correct residue appears among the first top-*k* predicted classes. Figure 2a shows the success rate as a function of the number of k predicted classes across training epochs. For all the models, we observe complete random performance at epoch zero, and as training progresses, the correct amino acid increasingly appears among the top-*k* predicted classes (i.e. at lower values of k). All the models exhibit a clear improvement trend in the success rate curves, supporting their suitability for further analysis.

#### 3.3.3 Variant confusion matrix and BLOSUM

While the primary objective of the model is to reconstruct masked residues, its most informative output is not simply whether it correctly recovers the native amino acid, but the distribution of scores assigned to all 20 candidate residues. Because the internal representations learned during self-supervised training are not directly interpretable, evaluating what the model has captured about structural constraints is inherently challenging. To probe these learned relationships, we computed the confusion matrix of masked residue predictions.

The confusion matrix provides insight into systematic substitution patterns by revealing which amino acids the model considers interchangeable within similar structural environments. Rather than viewing misclassifications as errors, these off-diagonal patterns can reflect underlying physicochemical similarities, such as shared size, charge, or hydrophobicity. Therefore, the confusion matrix serves as a proxy for the substitution tendencies implicitly learned by the model, allowing us to assess if its predictions align with known substitution tendencies rather than arising from random or frequency-driven biases.

The correlation between the model-derived substitution matrix and BLOSUM is shown in Fig. 2b across training epochs for the models trained on all structures for *n* = 16, 32, and 64 neighbors. Figure S2, available as supplementary data at *Bioinformatics* online, is the same plot for the models trained on wild-type structures for *n* = 16 and 32 neighbors.

For *n* = 16 neighbors, having an acceptable accuracy, loss, and success rate trajectories, the BLOSUM correlation matrix has the highest values of 0.26, which is a weak positive correlation (Fig. 2bi). This indicates that the model fails to fully capture biologically relevant information. Thus, 16 neighbors may be insufficient for correct residue prediction. In contrast, when using *n* = 32 neighbors the correlation matrinx with BLOSUM starts to show higher values around 0.33 (Fig. 2bii). As expected, the correlation between BLOSUM and the model-derived substitution matrix starts at epoch 0, indicating complete randomness. Then it starts to increase to reach correlation coefficient values above 0.3, in line with a moderate correlation with the BLOSUM matrix. This is a sign of biological relevance in model predictions. Figure 2ci presents the confusion matrices of the *n* = 32 model at epoch 80, with predicted residues on the x-axis and true labels on the y-axis for the validation set. The observed improvement in the correlation matrix from *n* = 16 to 32 neighbors motivated us to train another model with *n* = 64 neighbors. Figure 1b shows the accuracy and loss trajectories of this model. Since the input data is larger, this model converges more slowly, hence 700 epochs. The accuracy range of the model is also similar 16- and 32-neighbor models. Comparing the correlation with BLOSUM in Fig. 2biii however, shows an improvement in this value, 0.39, which is moderate correlation. This suggests that providing information from a larger number of neighboring atoms enables the model to learn more biologically meaningful relationships.

#### 3.3.4 Shuffled data model to study the model potential to overfit

**Figure 3:**
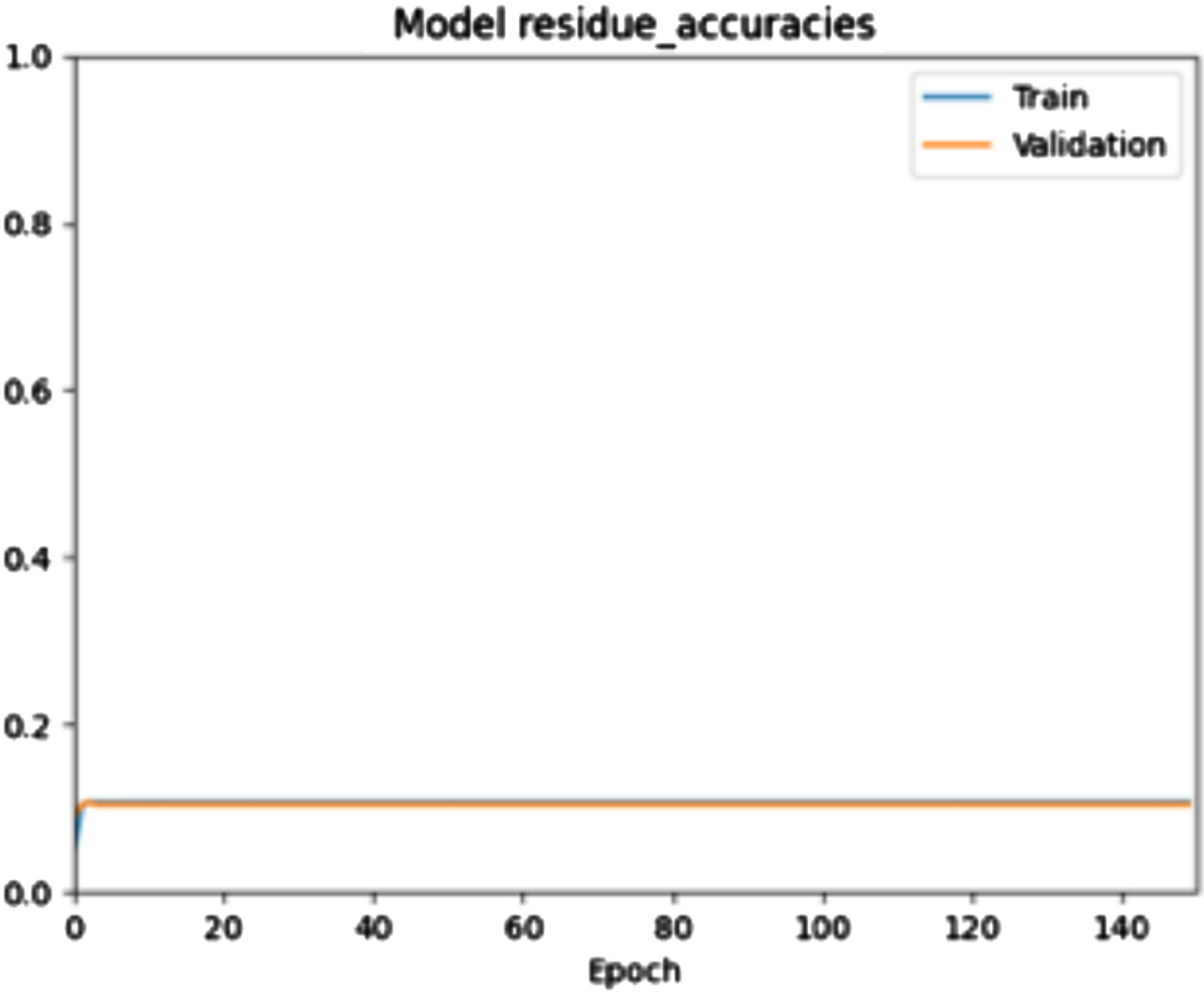
Shuffled label model loss. This model is trained on shuffled residue outputs to study the model’s capacity to overfit. The model is similar to the *n* = 32 model, except the shuffled output on the training set. This plot shows no sign of fitting on the training set, which shows the model is not memorizing the labels.

Overfitting is an important concern here, due to the high similarity of structural inputs across the training, validation, and test sets. To assess the model’s propensity to overfit, we trained the best-performing model configuration so far using shuffled residue labels in the training set. If the model were able to overfit, it would be expected to memorize the shuffled—wrong—residue identities in the training data. Figure 3 is the accuracy trajectory of the model trained on shuffled labels. This model was trained for 150 epochs. The accuracy remains constant around 0.1 even on the training set. This result indicates that this model does not memorize the training set labels and is therefore not overfitted. The hyperparameters used to train this model are identical to those used for our *n* = 32 model, which showed moderate correlation with BLOSUM matrix.

#### 3.3.5 Model evaluation on small GTPase

To validate if the model had simply memorized the data or it had truly learned the context, we generated masked partial graphs from the small GTPase group and evaluated the model’s performance in reconstructing this data. The model achieved an accuracy of 82.5% on the small GTPase dataset (Figs S3 and S4, available as supplementary data at *Bioinformatics* online). This illustrates that the model learned general rules of local atomic environments. The learned representation is therefore closer to a universal structure-conditioned amino acid predictor rather than a family-specific predictor.

#### 3.3.6 Resources

The 64-neighbor model was trained on a Linux workstation with 24 CPU cores and an NVIDIA RTX A5000 GPU with 24 GB of VRAM (CUDA 12.6).

The RAM required during training depends on the batch size and the *n* used for the *n*-neighbor graphs. For a 64-neighbor partial graph presentation, the model with a batch size of 512 takes ∼7900 s per epoch for training and ∼1500 s per epoch for validation. The model required ∼9.2 GB GPU memory during training on the NVIDIA RTX A5000 GPU.

## 4 Discussion

Understanding the functional consequences of mutations in protein kinases remains a fundamental challenge in molecular biology and biomedical research. Rather than approaching this challenge as a supervised classification task, we introduce a structure-based, self-supervised framework. Our approach learns residue compatibility directly from 3D protein environments using SE(3)-transformers. This method ensures that our model does not depend on inconsistent annotations of curated mutation effect, a significant concern given their high variability across databases. Instead, the model learns physicochemical and geometric constraints governing amino acid placement within experimentally resolved kinase structures. In this framework, mutation effect prediction arises because of learned structural constraints rather than from explicit supervision on pathogenicity labels.

Indeed, a challenge in evaluating models of mutation pathogenicity is the limited reliability and coherence of existing annotation datasets. Mutation labels aggregated from databases such as ClinVar, COSMIC, or population-scale resources often reflect conflicting interpretations: the classification of variants as pathogenic or benign frequently differs across databases and contexts. For binary classification tasks, such inconsistencies introduce substantial noise that can severely compromise the performance and interpretability of supervised models. Consequently, traditional benchmarking against annotated pathogenic/benign labels may underestimate the value of models that capture genuine structural constraints. Our results highlight the need for alternative evaluation strategies focused on biological consistency, such as agreement with substitution matrices, structural consistency, generalization ability to other families, or experimentally measured stability effects.

Importantly, the objective of the model is not merely to recover the native residue identity, which is already known, but to learn a probability distribution over amino acids compatible with a given structural environment. The scores assigned to alternative amino acids can therefore be interpreted as quantitative estimates of structural fitness. This probabilistic formulation can be viewed as analogous to an implicit energy model, in which residues assigned higher likelihood correspond to lower effective structural incompatibility within the local environment. The observed correspondence between model-derived substitution patterns and established evolutionary substitution matrices such as BLOSUM, as well as its ability to reconstruct masked resides in the small GTPase family with 82.5% accuracy, suggests that the model captures meaningful biochemical relationships rather than memorizing residue frequencies.

Several limitations should be considered. First, the model relies on experimentally determined structures, restricting coverage to kinases with available high-quality structural data and potentially biasing learning toward resolved and relatively stable conformational states. Second, local neighborhood representations, while computationally efficient, may fail to capture long-range allosteric effects that influence mutation outcomes. Third, structural compatibility does not fully determine pathogenicity, which may also depend on regulatory context, protein-protein interaction networks, and the broader cellular environment. Future work could extend this framework by incorporating conformational dynamics, ligand binding site information, predicted structures, or systems-level interaction data to more comprehensively model mutation effects beyond local structural compatibility.

Despite these limitations, the proposed framework offers several advantages. By avoiding supervised pathogenicity labels, the approach reduces dependence on potentially noisy and inconsistent annotations, and enables learning from large structural datasets. The equivariant architecture ensures geometrically and physically meaningful representations, allowing the model to generalize across orientations and conformations. Moreover, the framework is inherently extensible to other structurally conserved protein families beyond kinases. In contrast to large-scale models such as AlphaFold, ESM, and Boltz-2, which are trained on millions of sequences to learn broad protein representations or perform structure prediction, our framework is family-specific and focuses on learning fine-grained residue compatibility within local kinase structures. This enables a more localized and physicochemical view of mutational effects that is not explicitly captured by sequence-only or global structures-derived models.

Overall, this study demonstrates that self-supervised geometric deep learning provides a promising direction for interpreting genetic variation by learning the structural rules governing protein stability and compatibility. By reframing mutation effect prediction as a structure-conditioned learning problem rather than a label-driven classification task, our approach offers a complementary and structurally grounded perspective to traditional sequence- and annotation-based mutation prediction methods.

By providing position-specific estimates of structural compatibility, this approach may contribute to the interpretation of rare variants in clinical sequencing studies and support precision medicine efforts aimed at linking genetic variation to functional and therapeutic consequences.

## Supplementary Material

btag487_Supplementary_Data
