# Supplementary material for: Structure-conditioned self-supervised learning of residue interaction constraints in protein kinases for variant interpretation
Source: Bioinformatics. 2026 Aug 4;42(Suppl 2):btag487. doi: 10.1093/bioinformatics/btag487 (PMC13501329; doi:10.1093/bioinformatics/btag487)
Supplement: Supplementary file 1 [file btag487_supplementary_data.pdf]

Structure-Conditioned Self-Supervised Learning of Residue Interaction Constraints in Protein Kinases for Variant Interpretation

Shakiba Fadaei[1], Fanny S Krebs[1,2,*] and Vincent Zoete[1,2,*]

[1]Computer-Aided Molecular Engineering, Department of Fundamental Oncology, Ludwig Institute for Cancer Research, University of Lausanne, Lausanne, Switzerland, [2]SIB Swiss Institute of Bioinformatics, Lausanne, Switzerland.

Supplementary Information

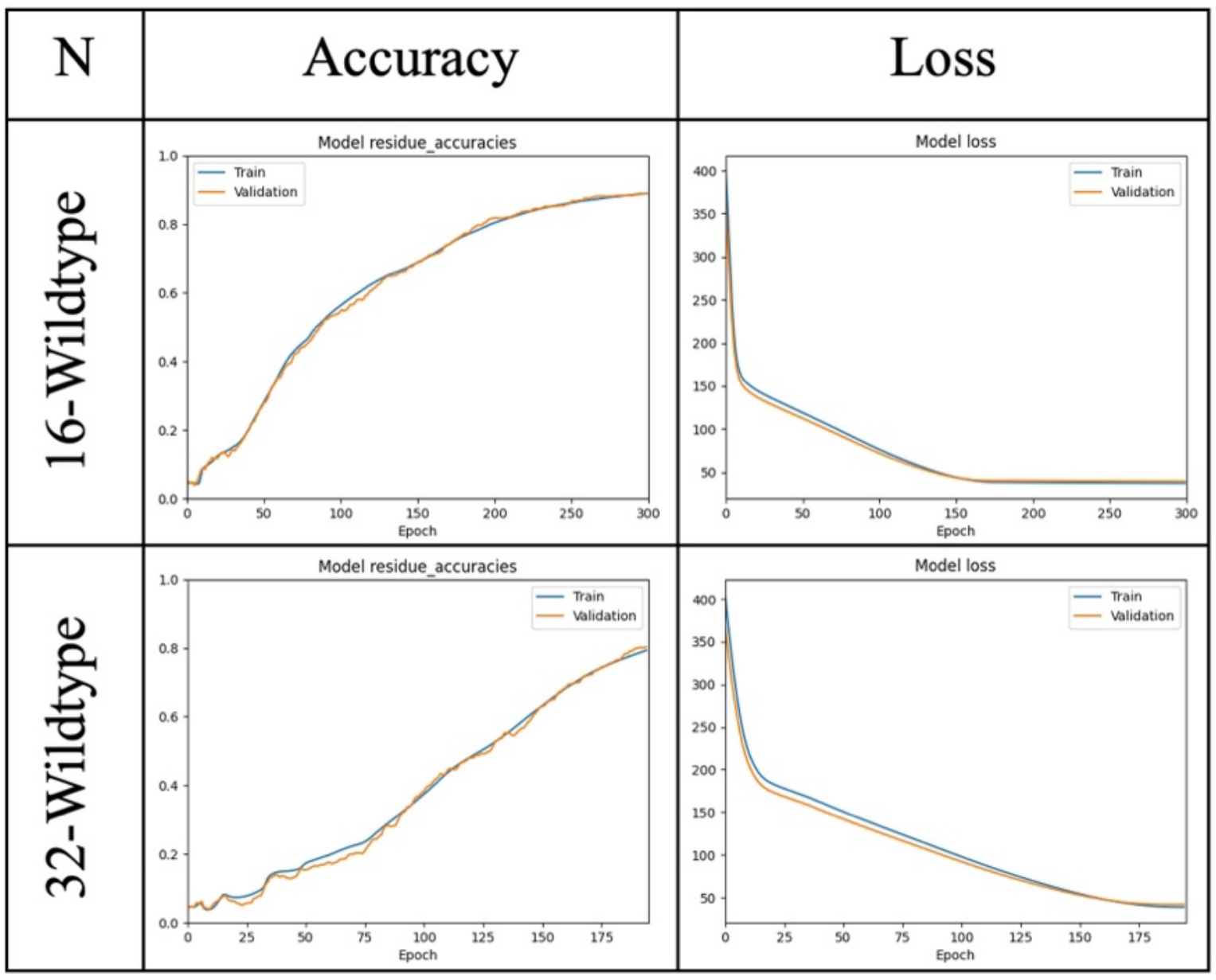

SI Figure 1. SE(3)-transformer model performance on wildtype structures. *The model accuracy and loss trajectory trained for 16-neighbors on only wildtype structures*

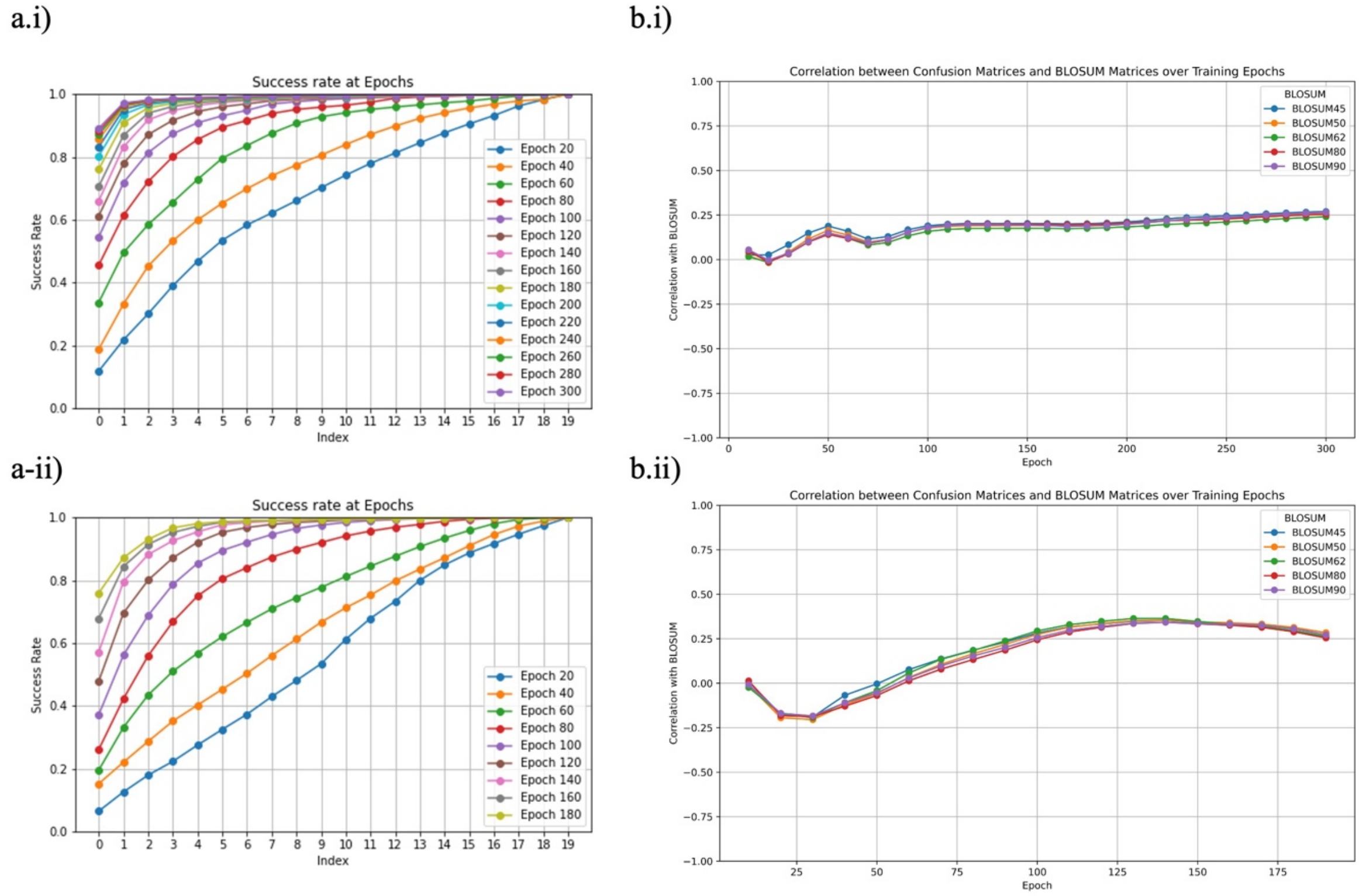

SI Figure 2. Model performance evaluation. a) *The success rate of the trained models during training epochs.* ***b)*** *Correlation between the confusion matrix and BLOSUM matrix .* ***i, ii)*** *are for 16- and 32-neighbors.*

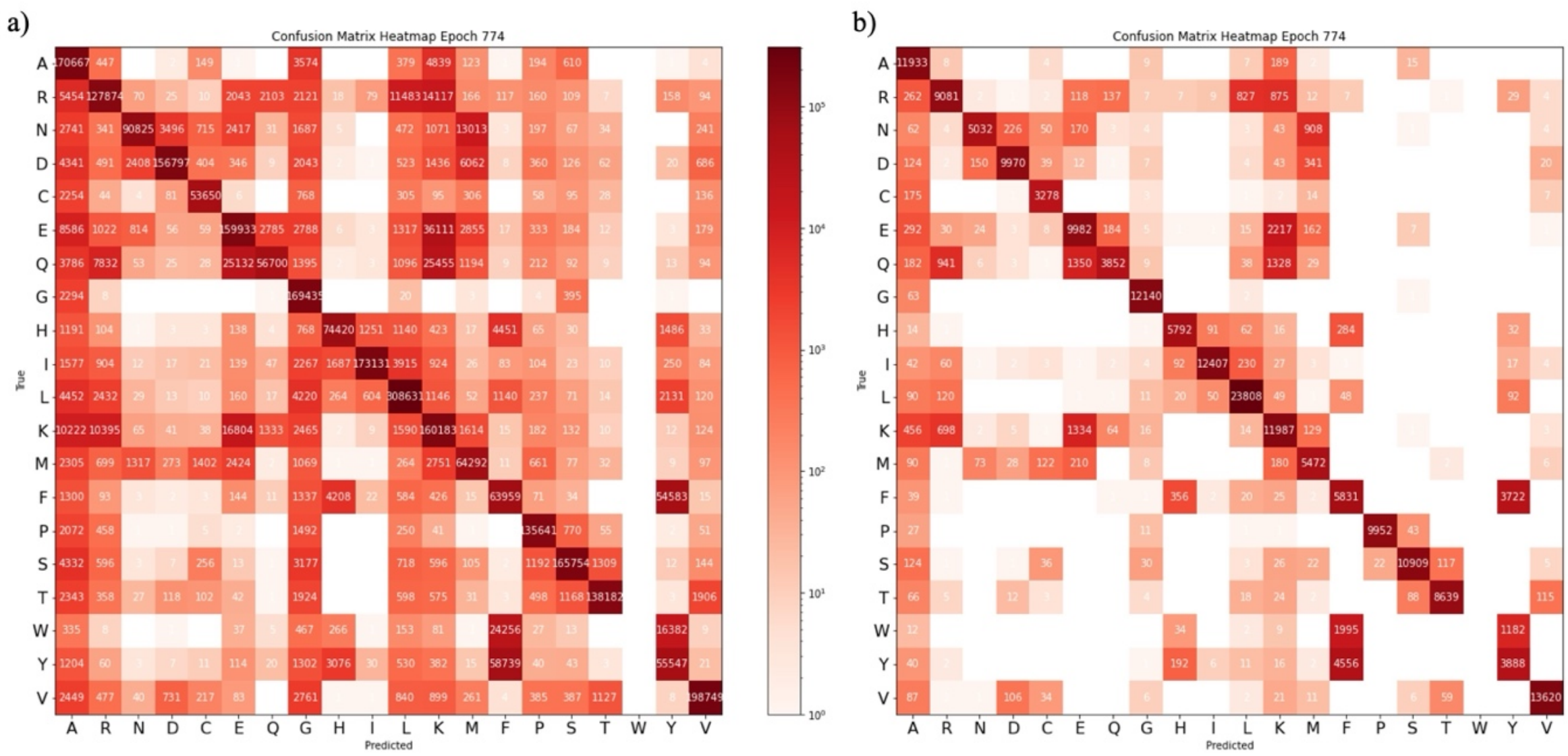

SI Figure 3. Model performance on masked partial graphs from small GTPase family. a) *The confusion matrix of the model on the small GTPase family at epoch 774.* ***b)*** *The model performance on the same epoch on the validation set of kinases. These matrices are strongly correlated, with a pearson correlation of 0.99591 but not numerically identical, due to more noise in the small GTPase group predictions. This is expected since the model was trained on kinases. This indicated good generalization, since the model predicts the residues given local context of another family correctly*

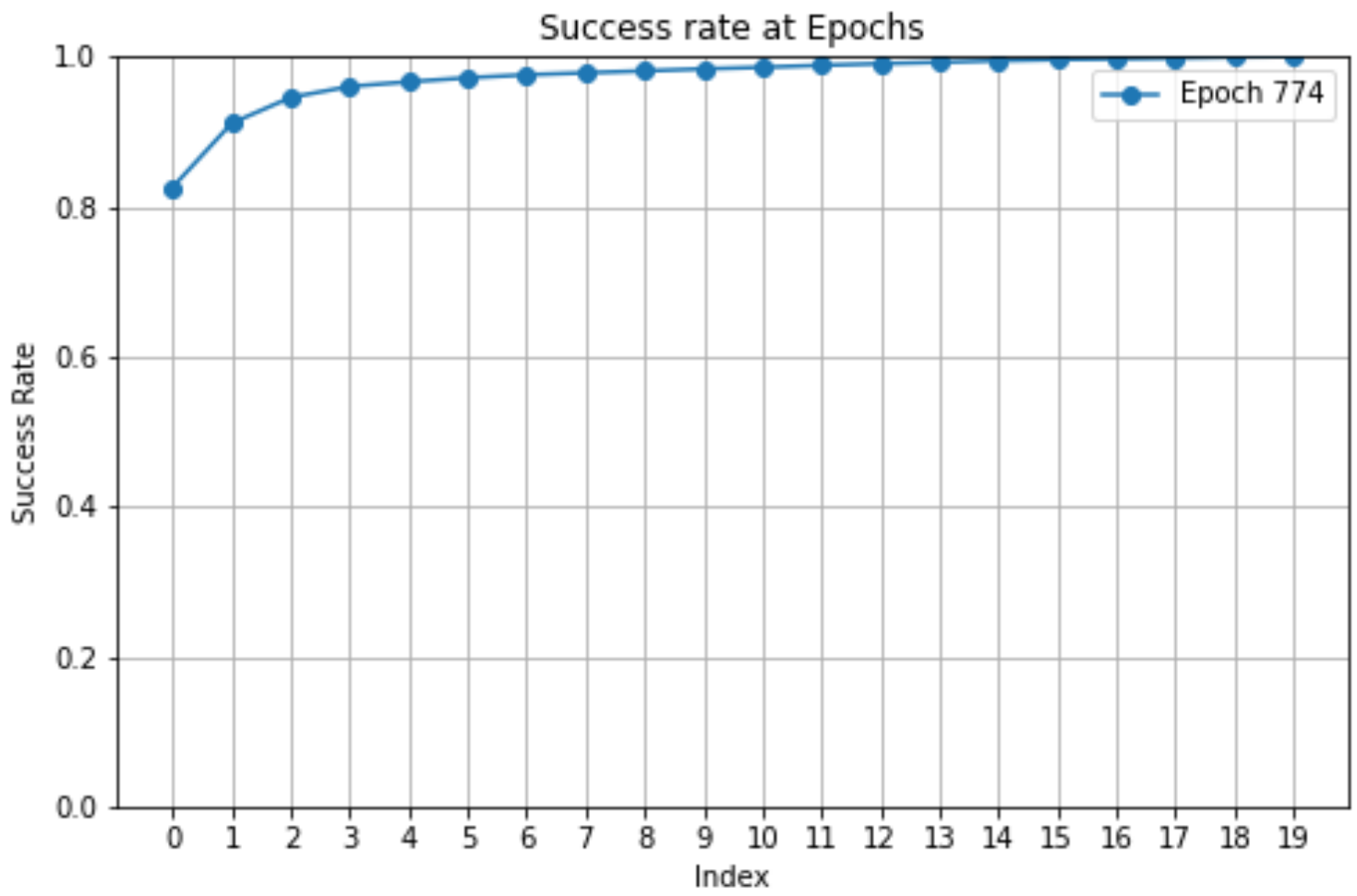

SI Figure 4. Model success rate on small GTPase masked residue prediction. *The model's first prediction success rate is usually called accuracy. As it is shown in this figure, the accuracy on masked small GTPase residue prediction is above 0.8, which indicates that the model is generalized.*
